# Preparation of Aluminum–Molybdenum Alloy Thin Film Oxide and Study of Molecular CO + NO Conversion on Its Surface

**DOI:** 10.3390/ma15062245

**Published:** 2022-03-18

**Authors:** Tamerlan T. Magkoev, Dzhamilya G. Mustafaeva, Vladislav B. Zaalishvili, Oleg G. Ashkhotov, Zaurbek T. Sozaev

**Affiliations:** 1 Laboratory of Adsorption Phenomena, Department of Condensed Matter Physics, North Ossetian State University, Vatutina 44-46, 362025 Vladikavkaz, Russia; dzhamilya79@yandex.ru (D.G.M.); ketroel@gmail.com (Z.T.S.); 2 Geophysical Institute—The Affiliate of Vladikavkaz Scientific Centre of the Russian Academy of Sciences, Markova 93a, 362002 Vladikavkaz, Russia; vzaal@mail.ru; 3 North Caucasian Mining and Metallurgical Institute, State Technological University, Nikolaev 44, 362021 Vladikavkaz, Russia; 4 Institute of Informatics, Electronics and Robotics, Kabardino-Balkarian State University, Chernyshevskogo 173, 360004 Nal’chik, Russia; oandi@rambler.ru

**Keywords:** adsorption, thin films, surface reaction, aluminum, molybdenum, surface alloy, carbon monoxide, nitric oxide, surface analysis techniques

## Abstract

Adsorption and interaction of carbon monoxide (CO) and nitric oxide (NO) molecules on the surface of bare Al-Mo(110) system and on that obtained by its in situ oxidation have been studied in ultra-high vacuum (base pressure: ca. 10^−8^ Pa) by means of Auger and X-ray photoelectron spectroscopy (AES, XPS), low energy electron diffraction (LEED), reflection–absorption infrared and thermal desorption spectroscopy (RAIRS, TDS), and by the work function measurements. In order to achieve the Al-Mo(110) alloy the thin aluminum film of a few monolayers thick was in situ deposited onto the Mo(110) crystal and then annealed at 800 K. As a result of Al atoms diffusion into the Mo(110) subsurface region and the chemical reaction, the surface alloy of a hexagonal atomic symmetry corresponding to Al_2_Mo alloy is formed. The feature of thus formed surface alloy regarding molecular adsorption is that, unlike the bare Mo(110) and Al(111) substrates, on which both CO and NO dissociate, adsorption on the alloy surface is non-dissociative. Moreover, adsorption of carbon monoxide dramatically changes the state of pre-adsorbed NO molecules, displacing them to higher-coordinated adsorption sites and simultaneously tilting their molecular axis closer to the surface plane. After annealing of this coadsorbed system up to 320 K the (CO + NO → CO_2_ + N) reaction takes place resulting in carbon dioxide desorption into the gas phase and nitriding of the substrate. Such an enhancement of catalytic activity of Mo(110) upon alloying with Al is attributed to surface reconstruction resulting in appearance of new adsorption/reaction centers at the Al/Mo interface (steric effect), as well as to the Mo d-band filling upon alloying (electronic effect). Catalytic activity mounts further when the Al-Mo(110) is in situ oxidized. The obtained Al-Mo(110)-O ternary system is a prototype of a metal/oxide model catalysts featuring the metal oxides and the metal/oxide perimeter interfaces as a the most active reaction sites. As such, this type of low-cost metal alloy oxide models precious metal containing catalysts and can be viewed as a potential substitute to them.

## 1. Introduction

In a search of low-cost heterogeneous catalysts, many studies are devoted to the model catalytic systems which do not contain precious metals [1]. One of the widely utilized approaches is alloying of transition and simple metals (d-metals and sp-metals) with the aim to achieve more d-band filling and thus get closer to noble metals with the completely filled d-band (electronic effect). At the same time, such an alloying produces additional adsorption/reaction sites at the interface of the components of the alloy (steric effect) [2,3]. These both effects are operative, for instance, in the case of tungsten and molybdenum carbides which demonstrate catalytic activity in CO reduction compared with that exhibited by noble metals [4,5]. In the case of FeCo alloy, changing the ratio of the components allows variation of the activity of the Fischer–Tropsch reaction, further suppressing the formation of carbides, thereby reducing the rate of catalyst degradation [6]. Another alloy as an efficient catalyst in hydrocarbon and carbon monoxide conversion is molybdenum nitride, featuring, for instance, high activity for selective oxidation of methane into alcohols [7,8]. As a demand of automotive exhaust reduction much attention is devoted to the studies of catalytic NO + CO conversion [9]. In this regard, it is shown, for example, that along with the noble metals—such as Rh, Pt, Pd, and Ir—the tungsten and molybdenum carbides may also be active catalysts for automotive exhaust neutralization [10,11].

Despite of quite extensive research in this field, there are lack of studies of the alloys containing metals of the center of the transition row (d5) with the sp-metals, such as, for instance, W or Mo with Al. At the same time, due to the specific electronic properties of half-filled d-band, and the excess of free charge in such a metal like Al, the d-sp hybridization would shift the center of the d-band down with respect to Fermi level, thus increasing its filling and, as a consequence, enhancing catalytic activity [9]. In line with this assumption are the previous results of increase in CO catalytic oxidation activity on Mo(110) upon alloying with boron, the element which is isoelectronic to Al [12].

In relation to this, the aim of the present study is to find out the mechanisms of possible conversion of carbon monoxide and nitric oxide molecules on the Al-Mo(110) and oxidized Al-Mo(110)-O systems.

## 2. Experimental

Measurements have been carried out in ultra-high vacuum (UHV base pressure: ca. 10^−8^ Pa) by means of AES (VG Scientific, Oxford, UK), XPS (VG Scientific, Oxford, UK), RAIRS (Nicolet, San Diego, CA, USA), TDS (Hiden-Analytical, Hamburg, Germany), LEED (Varian, Oxford, UK), and work function measurements in an Anderson mode with the aid of VG Scientific Escalab MII set-up. A single pass cylindrical mirror electron analyzer (modulation voltage: 2 V to detect the spectra in dN/dE mode) with a coaxial electron gun operating at a primary energy of 2 keV and a hemispherical deflector analyzer with monochromatized Al K_α_ irradiation (photon energy: 1486.6 eV) were used for AES and XPS measurements, respectively. The LEED studies were performed with the use of standard 4-grid electron retarding field optics with rear view of the patterns. The TDS spectra were registered via the quadrupole mass-spectrometer (Hiden Analytical) aligned along the surface normal, with the sample temperature sweep rate normally around 2 K/s. The quadrupole analyzer was adjusted for simultaneous detection of species with different molecular masses (*m*/*z*). The RAIRS measurements have been carried out using conventional infrared spectrometer Nicolet-Nexus 870 mounted around the UHV chamber in the way so that the beam source and the MCT detector cooled by liquid nitrogen were at the opposite sites of the sample. In this configuration the incidence and the detection angles of the p-polarized infrared beam were about 85 degrees (grazing incidence). The latter is necessary to provide maximal sensitivity to vibrational modes of the adsorbed molecules under consideration (CO, NO) which are aligned along the surface normal when adsorbed. The work function (φ) was measured by the Anderson method of contact potential difference (CPD) detection. For this, a low energy electron gun operating at a primary energy of a few electron-volts was mounted in the UHV chamber. The electron beam was directed to the sample with an energy sweeping between −10 eV to +10 eV to monitor the CPD between the sample and the cathode of the gun. In fact, only the relative change of the work function of the sample upon adsorption on its surface is detected in this mode. In order to obtain the absolute values of the work function the value of the φ of bare Mo(110) was set as 5.0 eV [13].

To obtain maximal atomic cleanliness of the Mo(110) substrate it was annealed at 1800 K in UHV chamber in an oxygen ambient at a partial pressure of 10^−5^ Pa to remove carbon and sulfur residual contaminations. Afterwards it was repeatedly heated to temperature up to 2600 K in ultra-high vacuum (base pressure: 10^−8^ Pa) until no contaminations were detected by AES. The Al film was in situ deposited onto Mo(110) via thermal evaporation of bulk aluminum of 99.9999% purity via the Knudsen cell. The deposition flux and thus the surface coverage of Al atoms (θ) was estimated via Mo MNV (188 eV) dN/dE Auger signal attenuation and the Mo(110) work function change upon Al deposition in submonolayer coverage range. The latter was performed as a control tool because it is known that there is direct correlation between the features of φ(θ) dependence and the surface concentration of adatoms [13,14]. Taking into account the proximity of Al and Mo atomic sizes, it was considered that the unity (one monolayer, ML) coverage (θ = 1) corresponds to surface concentrations of Al atoms of 1.5 × 10^15^ cm^−2^ [14]. At this coverage the atomic structure of Al film corresponds to Al(111) [14,15]. Upon the further alumina film thickness growth this structure holds up to 8–9 monolayers. This is due to the ordering effect of underlaying Mo(110) template. At higher Al film thickness the effect of Mo(110) support weakens, and, according to the featureless LEED pattern, the Al film surface becomes amorphous. In order to form the surface Al-Mo(110) alloy, initially a 2 ML thick aluminum film was in situ deposited onto Mo(110) substrate and then annealed at 800 K for 3 min. Such an alloy is stable during annealing up to 1400 K. The value of annealing temperature of 800 K was chosen since at this temperature the equilibrium state of Al-Mo surface alloy is achieved. It is noteworthy, that regardless of the initial thickness of Al overlayer deposited onto Mo(110), the surface structure of the formed Al-Mo alloy is (√3 × √3)R30° (see below). To obtain an adsorbed CO and NO molecules on the formed Al-Mo(110) surface alloy, the UHV chamber was filled by the corresponding gases of research grade quality up to partial pressure of 10^−5^–10^−6^ Pa. The gas exposure was set to be equal to unity (1 L) when the sample is exposed to gas ambient at partial pressure of 10^−4^ Pa for 1 s: 1 L = 10^−4^ Pa × s. To avoid uncertainty in interpretation of TDS signals of CO and N_2_ because of the same value of *m*/*z* = 28, as a nitric oxide an isotopically labelled ^15^NO was used. The sample could be cooled down to about 90 K via the liquid nitrogen filled reservoir attached to the mounting wires. To form the alloy oxide the Al-Mo(110) sample was exposed in situ to molecular oxygen admitted into the vacuum chamber to achieve exposure of 1500 L while holding the sample temperature at 700 K. Adsorption of CO and NO on the formed oxide was performed after cooling it down to 90 K.

## 3. Results and Discussion

**Figure 1:**
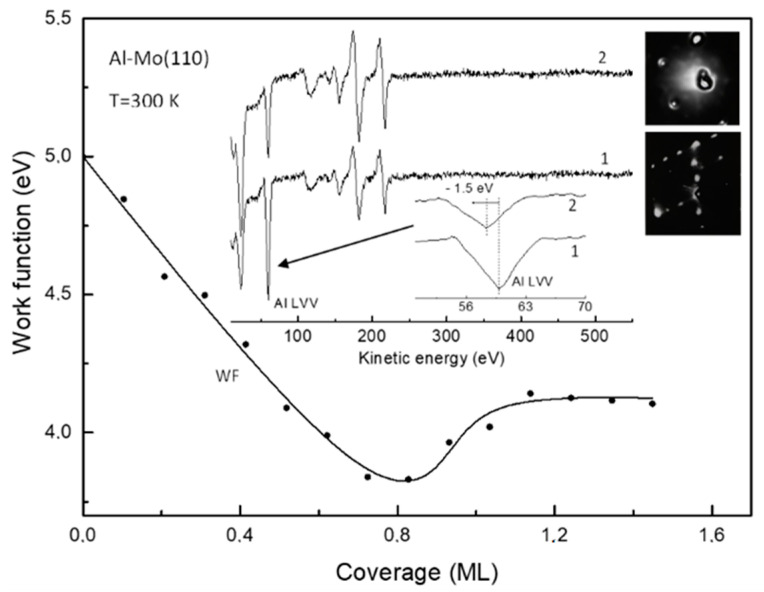
The work function versus coverage plot upon aluminum adsorption on Mo(110) held at room temperature. The Auger spectra and the LEED patterns of Al films of 2 ML thick prior and after thermal annealing at 900 K for 3 min (spectra 1 and 2, respectively) are shown in an inlay.

**Figure 2:**
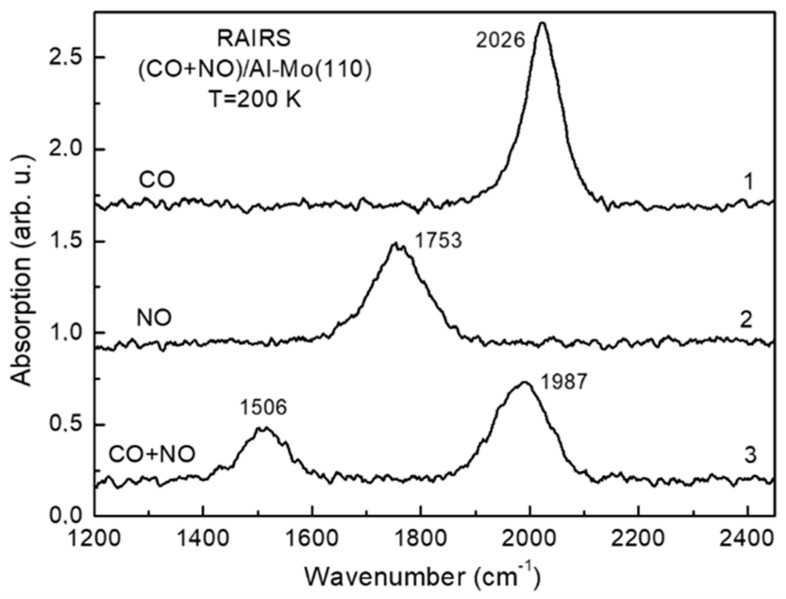
The Al-Mo(110) adsorbed CO and NO RAIRS spectra in the case of separate (spectra 1 and 2) and cooperative (spectrum 3) adsorption at a substrate temperature of 200 K. The spectrum 3 is for post-adsorption of CO molecules on the alloy surface precovered by the NO. In all cases both CO and NO exposure is 20 L.

**Figure 3:**
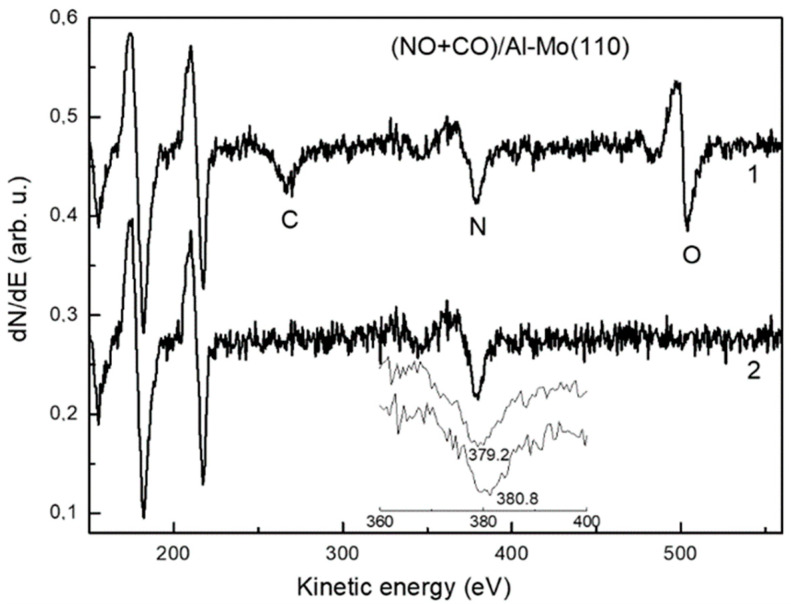
Transformation of the Auger spectra of (CO+NO)/Al-Mo(110) upon annealing of the initially formed system at a substrate temperature of 200 K (spectrum 1) to 380 K (spectrum 2). The expanded nitrogen N KLL region is also shown to demonstrate the Auger shift upon annealing.

The work function versus coverage plot φ(θ) upon deposition of Al atoms in submonolayer region onto Mo(110) held at room temperature is shown in Figure 1. In lines with the common view of interpretation of such curves, the initial work function decrease is due to the polarization of the chemisorption Al-Mo charge towards the substrate (electropositive adsorption). Moreover, the overall shape of the φ(θ) curve, featuring a minimum followed by a plateau, is additional evidence for this assumption: All alkali and alkaline earth metals, which are always electropositive when adsorbed on refractory metal crystals, have φ(θ) plots of the same shape [16]. It is noteworthy, that despite of the quite extensive research of adsorption of metal atoms on the metal substrates, the studies of Al on Mo(110) and other metals are lacking. In of the such previous studies of Al-Mo(110), the authors report almost the same φ(θ) plot [14]. With account of the Helmholtz equation Δφ = 4πθeμ, where e and μ are the electron charge and the dipole moment, respectively. The initial dipole moment of the Al-O polarized chemisorption charge at zero coverage limit (θ → 0) is 0.190 D, which is close to that (0.182 D) reported in reference [13]. Regarding the realized morphology of the aluminum film deduced from the Auger uptake curves and the LEED patterns, the growth mode is layer-by-layer with the formation of Al(111) structure [13,14]. When the aluminum film on Mo(110) is annealed, the Al atoms diffuse into the subsurface region with the formation of Al-Mo(110) surface alloy [14]. This results in the increase in the work function from 4.15 eV, characteristic for Al as-deposited film, to 4.4 eV. As has been demonstrated previously, annealing of Al-Mo(110) adsorbate systems leads to interdiffusion of the components and the formation of the alloy with twelve equilibrium phases with the dominant MoAl_12_ stoichiometry [17,18]. Other studies also predict formation of wealth of thin films alloys Al_x_Mo_100−x_ in a wide range of x values [19]. According to the LEED pattern, shown in Figure 1, the structure of as-deposited aluminum film at θ = 2 ML corresponds to Al(111). After annealing at a temperature of 800 K the Al Auger intensity dramatically drops (Figure 1). The latter is evidence of diffusion of Al from the surface into the bulk of the substrate. Decrease in Al Auger peak intensity is accompanied by its shift to lower energy by 1.5 eV, which is evidence of chemical interaction between Al and Mo. According to LEED, alloying leads to surface reconstruction with atomic structure (√3 × √3)R30°. The similar pattern was also observed upon annealing of Al film on Ru(1000) by Campbell and Goodman and was interpreted as a slightly distorted close-packed hexagonal structure of Al_2_Ru alloy [20]. This alloy of orthorhombic structure features stacking sequence of hexagonal planes, consisting of ordered mixture of Al and Ru atoms, corresponding to Al coverage of 2/3. An excess aluminum diffuses into the bulk upon annealing. As such, due to the similarity of observed structures in the present study and in reference [20], and the very close values of lattice constants of Mo and Ru crystals, one can expect formation of the similar surface alloy (Al_2_Mo) and in this case. This is corroborated by the above-mentioned fact of work function increase from 4.15 eV to 4.4. eV which, according to φ(θ) plot, corresponds to coverage of 0.55 ML. The latter is in rather close agreement with the aluminum content in Al_2_Ru surface alloy—2/3 [20]. The electronic state of the transition metal dramatically changes upon alloying with the simple metal. The dominant effect is increase in the d-band filling and, as a consequence, an effective downshift of the d-band center with respect to the Fermi level [2,9]. The latter (electronic effect), along with the transformation of the atomic structure/morphology of the substrate (steric effect) has a significant impact on the molecular adsorption and conversion. In terms of determining the fundamental elementary steps of catalytic rection the most widely utilized probing species are CO and NO molecules, as they prototype the main molecular orbitals [10,21]. In relation to this, such an approach is also used and in the present study. Figure 2 features the RAIRS spectra of CO and NO adsorbed separately on an Al-Mo(110) alloy cooled down to 200 K. Both spectra consist of a single line centered at wavenumber 2026 cm^−1^ and 1753 cm^−1^ for CO and NO, respectively. These lines correspond to intramolecular stretch vibration. In this regard, an apparent difference of Al-Mo(110) alloy from both bare Mo(110) and Al(111) is that in the latter cases no bands are detected upon CO and NO adsorption. This is obviously due to the fact that, unlike the Al-Mo(110) alloy, the CO and NO molecules dissociate on the surface of bare Al(111) and Mo(110) substrates [21]. Such a dramatic adsorption/reaction pathway shift upon alloying is sought to be due to combination of electronic and steric effects. Similar effect was observed by Ren et al. who have found suppression of dissociation channel of NO on molybdenum when it is alloyed with carbon [22]. Similarly, the same holds for alloying of Pd(111) with aluminum [23]. As mentioned above, the accepted idea is that this is due to higher degree of d-band filling for the alloy than for the bare transition metal, on one hand, and to the effective downshift of the d-band center against the Fermi level—on the other. In lines with the Blyholder model of molecular chemisorption [24], this electronic effect results in weakening of charge back-donation from the substrate d-band to the CO and NO 2π * antibonding orbitals. The wavenumber of stretch vibration of both CO and NO correspond to upright adsorption geometry with the nitrogen and carbon ends of the molecules bound to the substrate [25]. Higher coordinated adsorption sites such as bridge and hollow sites are generally characterized by lower wavenumbers than shown in Figure 2 [25]. Therefore, it seems reasonable to assume that the CO and NO molecules do not bound to the surface via these adsorption sites, presumably because they are blocked by alloying. As has been demonstrated by Campbell and Goodman [20], these higher coordinated sites are occupied by Al upon alloying. Along with this steric effect, one cannot exclude the effect of transformation of electronic sate of the substrate upon the molecular adsorption. Similar electronic effect was observed, for instance, when Pt(111) has been alloyed with Ge [26]. Despite of the very low content of Ge (ca. 5%), adsorption behavior of CO and NO molecules on the surface of the alloy dramatically changes relatively to that on bare Pt(111). When the CO and NO molecules are cooperatively adsorbed on the surface of the Al-Mo(110) alloy (CO at an exposure of 20 L is adsorbed on 20 L NO precovered substrate) there are new features in the RAIRS spectrum which are not present in the case of separate adsorption (Figure 2, spectrum 3). This (CO + NO)/Al-Mo(110) system is characterized by the Auger spectrum, shown in Figure 3, spectrum 1. This spectrum consists of Mo, Al, O, N, and C lines with no other species detected pointing at the atomic cleanliness of the system under consideration. The feature of the RAIRS spectra is that the wavenumber value for both CO and NO in the case of cooperative adoption is red-shifted by 250 cm^−1^ and 40 cm^−1^ for NO and CO, respectively compared to the case of separate CO and NO adsorption. The NO wavenumber of 1506 cm^−1^, according to the accepted viewpoint [25], corresponds to the bridging and/or hollow site adsorbed molecules, whereas that of CO (2026 cm^−1^)—to atop site molecules. The lower intensity of the vibrational bands of both molecules in the case of cooperative adsorption is likely due to the tilting of the molecular axis closer to the surface plane (tilted adsorption geometry). However, for NO the situation is more dramatic as the wavenumber value shifts much more significantly (by 250 cm^−1^) than for CO, for which the wavenumber shifts only by 40 cm^−1^. Such a difference is due to the fact that the NO not only tilts closer to the surface, but also shifts from atop adsorption site for separate adsorption to higher coordinated center, such as bridge or hollow site, for cooperative adsorption. For separate adsorption of NO on the Al-Mo(110) surface the preferred adsorption site is atop, because the corresponding RAIRS spectrum consists of only one absorption band at high wavenumber (1753 cm^−1^) (Figure 2, curve 2). However, when the CO molecules are post-adsorbed on NO precovered substrate, the latter are displaced from initially occupied atop sites to bridge and/or hollow sites with simultaneous tilting their molecular axis closer to the surface plane. Becoming effectively closer to the surface, the molecules are stronger affected by both Mo and Al, thus enhancing charge backdonation from the substrate to the NO antibonding 2π* orbital of the molecule. This causes wreaking of the molecular bond which is manifested in the observed wavenumber red shift. It is noteworthy that the similar effect of displacement of NO molecules by subsequent CO adsorption was also observed for the Ni(111) bulk crystal and Ni/MgO(111) metal/oxide substrate [27,28]. Upon slight annealing of the adsorbed system up to 350 K the RAIRS results in featureless spectrum with the corresponding Auger spectrum shown in Figure 3 (spectrum 2). It is seen that the Auger lines of carbon and oxygen are no longer detected, whereas that of nitrogen remains almost unchanged with except of a shift to higher energy by 1.6 eV.

**Figure 4:**
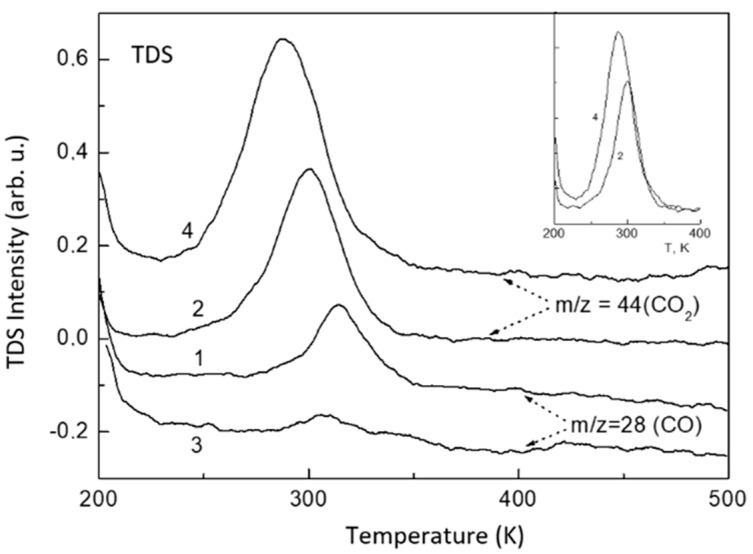
The TDS spectra of CO and CO_2_ of (CO + NO) coadsorbed system on Al-Mo(110) (spectra 1 and 2) and Al-Mo(110)-O (spectra 3 and 4). Exposure of both pre-adsorbed NO and post-adsorbed CO is 20 L at a substrate held at 200 K. For convenience, the inlay demonstrates comparison the CO_2_ TDS intensity for Al-Mo(110) (spectrum 2) and for Al-Mo-O (spectrum 4).

Such an observation indicates that annealing results in molecular conversions. To study such an effect in more detail the TDS measurements have been performed. The corresponding spectra are shown in Figure 4. The only features detected are the desorption lines corresponding to CO (*m*/*z* = 28) and CO_2_ (*m*/*z* = 44). The former species is reasonable to assign to thermodesorption of a certain part of CO molecules adsorbed on the surface at low temperature, whereas the latter—to oxidation of the remaining CO, presumably by NO (CO + NO → CO_2_ + N). This effect resembles that characteristic for precious metal containing catalysts, such as Pd, Rh, Pt [29]. Based on the above data, as a step of this reaction one would assume dissociation of NO molecules. As seen in RAIRS spectrum 3 (Figure 2), the NO molecule being in a tilted geometry bound to high coordinated site, is in pre-dissociation state, as it is affected by enhanced influence of both Al and Mo, the materials that adsorb NO dissociatively. This is in lines with previous findings that it is NO, not CO, that dissociates on other molybdenum- and tungsten-based alloys [10,11]. As such, the oxygen released after dissociation of NO reacts with CO which is in a favorable tilted geometry to form CO_2_. The latter desorbs into the gas phase as seen by the corresponding TDS spectrum (Figure 4). On the other hand, the nitrogen atoms after NO reduction, instead of their expected recombination to form N_2_ and desorb into the gas phase, remain at the surface. This is evidenced by the Auger spectrum of N KLL shown in an inlay of Figure 3. An observed Auger shift by 1.6 eV is a definite indication that there is chemical bonding between the nitrogen and the alloy. The as-formed ternary Al/Mo/N alloy may be an even more active catalyst in lines with the previous studies demonstrating high activity of aluminum and molybdenum nitrides for CO conversions [7,8,30,31]. At the same time, the oxygen released by NO dissociation mainly oxidizes the CO, not Al or Mo. This may be due to competition of N and O for the adsorption site, the effect most strikingly manifested in the case of substitution of oxygen by the nitrogen and formation of AlN even in aluminum oxide, as demonstrated in reference [32].

The reason of dramatic transformation of molecular adsorption and reaction properties under alloying of Mo(110) with Al is two-fold. On one hand, it is due to atomic reconstruction of the surface, and to the transformation of the electronic state of the alloy surface as a whole—on the other. As mentioned above, formation of a surface atomic structure of the alloy corresponding to (√3 × √3)R30° symmetry, results in appearance of lower coordinated sites compared to bare Mo(110), in addition to new adsorption sites at the interface between Al and Mo atoms. These centers are preferential NO adsorption sites as follows from the low value of the NO wavenumber 1506 cm^−1^ (Figure 2). Moreover, according to DFT calculations, the downshift of an effective center of the d-band upon alloying has a significant impact on the redistribution of the intramolecular charge of molecules upon adsorption [33].

**Figure 5:**
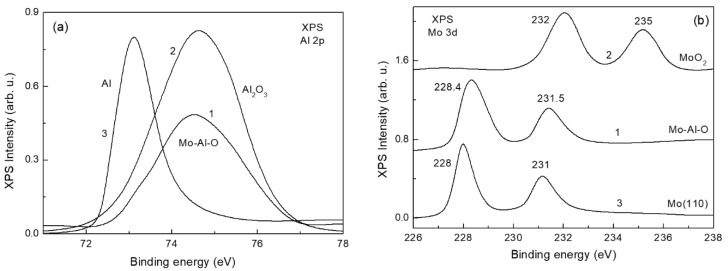
(**a**) The X-ray photoelectron spectra of Al 2p for Al-M-O alloy (spectrum 1), 10 nm thick alumina film on Mo(110) (spectrum 2), and 8 nm thick aluminum film on Mo(110) (spectrum 3). (**b**) The Mo 3d XPS lines for Al-Mo-O (spectrum 1), the MoO_2_ obtained by oxidation of the Mo(110) (spectrum 2), the bare Mo(110) (spectrum 3).

Oxidation of the Al-Mo(110) alloy results in further modification of adsorption and reaction properties of molecules. To assess the atomic state of the oxide the XPS measurements have been carried out. The corresponding spectra for an alloy oxide obtained by in situ oxidation of Al-Mo(110) held at 800 K in an oxygen ambient at a partial pressure of 10^−5^ Pa are shown in Figure 5a,b (spectra labelled by 1). For more unambiguous interpretation of these spectra, they are compared with the ones of aluminum and molybdenum oxides. The aluminum and molybdenum oxides (Al_2_O_3_ and MoO_2_) were obtained by well-known procedure of in situ oxidation, first—of the deposited aluminum film, the second—of the bare Mo(110) bulk crystal [34,35]. These spectra (labelled by 2), along with the spectra of metallic Al and Mo (labelled by 3), are compared with the spectra of the alloy oxide (Figure 5a,b). As seen, the Al 2p XPS spectra in an oxide and in the alloy are almost identical (spectra 1 and 2, Figure 5a), whereas the state of Mo in an alloy considerably differs from that in MoO_2_ (spectra 1 and 2, Figure 5b). At the same time, the vicinity of Mo 2p photoelectron lines in an alloy and in metallic Mo points that, unlike Al, the state of Mo atoms in the alloy oxide is close to that in metallic molybdenum. Nevertheless, the observed slight blue shift and broadening of the photoelectron lines (spectra 1 and 3, Figure 5b) are evidence that there is a certain transformation of an electron state of Mo atom upon oxidation of the alloy, although to lower extent than for Al. Thus, oxidation of the Al-Mo(110) alloy to an equilibrated state results in almost total oxidation of Al to alumina state, on one hand, and in rather insignificant change of the state of Mo—on the other. As follows from LEED, oxidation of the initially ordered Al-Mo(110) alloy surface leads to its disordering with no diffraction spots detected. In terms of ternary compounds one can assume formation of surface non-stoichiometric molybdenum aluminate Mo_1−x_·nAl_2_O_3_ [36], featuring the state of Al and Mo atoms qualitatively similar to that in the Al-Mo(110) alloy oxide under consideration.

Behavior of CO and NO molecules on an alloy oxide surface quite significantly changes compared to bare alloy. The evidence is the transformation of (CO + NO) TDS spectra upon transition from the alloy (spectra 1 and 2) to the alloy oxide (spectra 3 and 4) (Figure 4). In the latter case, the CO_2_ signal intensity (spectrum 4) grows compared to spectrum 2 for Al-Mo(110), accompanied by an almost complete disappearance of CO desorption signal (spectrum 3 versus spectrum 1). These facts definitely indicate that the catalytic efficiency for CO + NO conversion on an Al-Mo(110) alloy oxide is notably enhanced against the bare alloy, enhanced to the extent of almost total CO consumption. Such a high efficiency of the alloy oxide may be related to that the latter can be viewed as a prototype of Mo/Al_2_O_3_ metal/oxide supported catalyst of which high activity is attributed to the features of the supported metal particle (Mo) being in contact with the oxide (Al_2_O_3_), and the metal-oxide perimeter interface [37,38]. Taking into account the above results, one can assume that the selectivity and activity of (CO + NO) conversion over the Al-Mo(110) alloy surface and its oxide are quite similar to that characteristic for precious metal containing catalysts. Moreover, activity and selectivity can be tuned via changing of the stoichiometry of the multicomponent compound. Thus, these types of compounds may be viewed as potential low-cost substitutes for precious metal containing catalysts. Compared to transition metal carbides that are also often viewed as substitutes to noble metals [4,5], the present substrate is advantageous since it does not require the noble metal overlayer to gain the catalytic activity, which is the case for transition metal carbides [39].

## 4. Conclusions

Formation of aluminum/molybdenum surface alloy (Al-Mo(110)), its oxide, and investigation of the adsorption and interaction of CO and NO molecules on their surface have been performed in ultra-high vacuum (base pressure: 10^−8^ Pa) by means of a complimentary set of surface analysis techniques. The Al-Mo(110) alloy of MoAl_2_ stoichiometry and hexagonal surface atomic structure is formed when a few monolayer thick aluminum film deposited onto Mo(110) crystal is annealed at 800 K. Regarding its surface adsorption properties, the carbon monoxide (CO) and nitric oxide (NO) molecules adsorb molecularly at a substrate held at a temperature of 200 K. This contrasts with the bare Mo(110) and Al(111) substrates which both adsorb CO and NO molecules dissociatively. The NO molecules adsorbed on the Al-Mo(110) alloy surface are dramatically affected by post-adsorption of the CO molecules: They are displaced from initially occupied atop to bridge and/or hollow sites with simultaneous tilting of their molecular axis closer to the surface plane. There are several steps to which the heating of the system leads: CO to CO_2_ oxidation, NO reduction, nitriding of the surface, and CO and CO_2_ desorption into the gas phase. In this sense the catalytic behavior of the alloy resembles that exhibited by precious metal containing catalysts. Such a catalytic activity of the alloy is attributed to the surface atomic reconstruction, on one hand, and to the modification of the electronic structure of the substrate upon alloying, on the other. The former leads to the formation of active undercoordinated adsorption/reaction sites, the latter—to increase in the d-band filling of transition metal matrix, thus obtaining the alloy closer to noble metals, which are active catalysts. Catalytic activity increases further when the Al-Mo(110) alloy is oxidized. This is due to appearance of a new undercoordinated sites because of the surface reconstruction under oxidation, metal/oxygen interface sites, transformation of the local electron state of both Al and Mo, and the substrate as a whole. At least in terms of (CO + NO) conversion, Al-Mo(110) alloy and its oxide can be viewed as a potential low-cost substitute to noble metal containing catalysts.

## Data Availability

Data available on request due to privacy restrictions. The data presented in this study are available on request from the corresponding author. The data are not publicly available due to institutional internal regulations.
